# Roles of Polo-like kinase 3 in suppressing tumor angiogenesis

**DOI:** 10.1186/2162-3619-1-5

**Published:** 2012-04-18

**Authors:** Dazhong Xu, Qi Wang, Yongping Jiang, Yanxi Zhang, Eleazar Vega-SaenzdeMiera, Iman Osman, Wei Dai

**Affiliations:** 1 Department of Environmental Medicine, New York University Langone Medical Center, 57 Old Forge Road, Tuxedo, NY 10987, USA; 2 Memorial Sloan-Kettering Cancer Center, New York 10023, USA; 3 Biopharmaceutical Research Center, Chinese Academy of Medical Sciences & Peking Union Medical College, Suzhou, China; 4 Department of Dermatology, New York University Langone Medical Center, 522 First Avenue, New York, NY 10016, USA

**Keywords:** Plk3, Tumor angiogenesis, Tumor suppression, HIF-1α, PTEN

## Abstract

Angiogenesis is essential for promoting growth and metastasis of solid tumors by ensuring blood supply to the tumor mass. Targeting angiogenesis is therefore an attractive approach to therapeutic intervention of cancer. Tumor angiogenesis is a process that is controlled by a complex network of molecular components including sensors, signaling transducers, and effectors, leading to cellular responses under hypoxic conditions. Positioned at the center of this network are the hypoxia-inducible factors (HIFs). HIF-1 is a major transcription factor that consists of two subunits, HIF-1α and HIF-1β. It mediates transcription of a spectrum of gene targets whose products are essential for mounting hypoxic responses. HIF-1α protein level is very low in the normoxic condition but is rapidly elevated under hypoxia. This dramatic change in the cellular HIF-1α level is primarily regulated through the proteosome-mediated degradation process. In the past few years, scientific progress has clearly demonstrated that HIF-1α phosphorylation is mediated by several families of protein kinases including GSK3β and ERKs both of which play crucial roles in the regulation of HIF-1α stability. Recent research progress has identified that Polo-like kinase 3 (Plk3) phosphorylates HIF-1α at two previously unidentified serine residues and that the Plk3-mediated phosphorylation of these residues results in destabilization of HIF-1α. Plk3 has also recently been found to phosphorylate and stabilize PTEN phosphatase, a known regulator of HIF-1α and tumor angiogenesis. Given the success of targeting protein kinases and tumor angiogenesis in anti-cancer therapies, Plk3 could be a potential molecular target for the development of novel and effective therapeutic agents for cancer treatment.

## Introduction

When tumors grow to a certain size, a severe hypoxic microenvironment develops within the tumor mass [1]. Simple diffusion of oxygen and nutrients becomes insufficient to meet the demand of fast growing tumors [1]. It is believed that tumor size generally will not exceed 1 centimeter in diameter without the support of angiogenesis [2]. Tumor angiogenesis is a process leading to formation of vasculature that supplies the growth of solid tumors [1,2]. Logically, tumor angiogenesis tends to be more pronounced in aggressive and malignant tumors [3]. Since the initial discovery of tumor angiogenesis four decades ago [4], tumor angiogenesis has emerged as an important target for cancer therapy [1,2,5-7]. Many inhibitors of tumor angiogenesis have been developed and used in clinical application or is in the pipeline leading to clinics [1,2,6,7]. Since highly vascular tumors tend to have much higher potential for metastasis [8], these inhibitors of angiogenesis may have the added value of blocking tumor metastasis, thus making them more effective for cancer treatment.

A primary consequence of increased tumor size is hypoxia, a result of oxygen restriction in the tumor mass. Hypoxia triggers tumor angiogenesis by activating the cellular hypoxia response pathways [1]. Located in the center of these pathways are the hypoxia inducible factors (HIFs) including the well characterized HIF-1 [1,9-11]. HIF-1 is a transcription factor that promotes transcription of a series of genes such as vascular endothelial growth factor (VEGF) that are critical for the cellular hypoxic response [1,5].

HIF-1α is the inducible subunit of the HIF-1 transcription factor, whose protein level can be dramatically induced upon exposure to hypoxia [1,9-11]. The cellular level of HIF-1α protein is regulated primarily at the post-translational level [1,9-11]. The HIF-1α gene is constitutively transcribed and translated [1,9-11]. Under normoxic conditions, HIF-1α protein is hydroxylated by prolyl hydroxylases (PHDs), recognized and polyubiquitinated by and degraded by a proteosome-dependent mechanism [1,9-11]. Hypoxia greatly stabilizes HIF-1α because of a reduced activity of PHDs due to low oxygen tension [1,9-11]. In addition to hydroxylation, phosphorylation by protein kinases also regulates HIF-1α stability and/or localization [12-17]. Compared to hydroxylation, the significance of HIF-1α phosphorylation in regulating its stability is far less appreciated. Most recent studies have demonstrated that Plk3 is an endogenous kinase of HIF-1α that regulate its stability [17]. In addition, Plk3 phosphorylates and stabilizes PTEN phosphatase [18], which can in turn contribute to HIF-1α stability by suppression of the PDK/Akt signaling axis. Supporting this, *PLK3 *knock-out murine embryonic fibroblasts (MEFs) display significantly enhanced expression of HIF-1α and tumor angiogenesis in response to hypoxia [17,19]. In this review, we will discuss significance of Plk3 in HIF-1α regulation, tumor angiogenesis, and its potential as a therapeutic target for cancer treatment.

## Polo-like kinase 3

**Figure 1:**
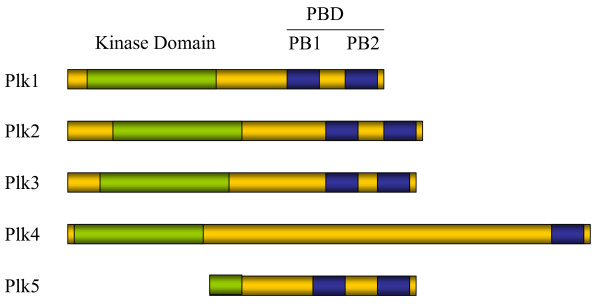
**Domain structures of human Plks**. All Plks have a highly conserved kinase domain and a polo box domain (PBD). Plk 1-3 and 5 have two polo boxes (PB) while Plk4 has one discernable PB. Human Plk5 has a truncated kinase domain.

Polo-like kinases (Plk) are named after *Drosophila *Polo, the founding member of a family of evolutionarily conserved protein serine/threonine kinases. Polo is involved in the regulation of mitosis and meiosis [20,21]. The mammalian Plk family consists of 5 members: Plk1, Plk2, Plk3, Plk4 and Plk5 [21,22]. These proteins share significant amino acid sequence homology to Polo and to one another [21]. Structurally, all Plks are comprised of a highly conserved kinase domain at the amino-terminus and a Polo box domain (PBD) at the carboxyl-terminus [21,23] (Figure 1). However, human Plk5 has a truncated kinase domain that lacks kinase activity [22]. PBD is believed to be important for the subcellular localization and the substrate recognition by these kinases [21,23]. The biological functions of mammalian Plks are more diverse than those of their counterparts in lower eukaryotes [21]. Among the Polo kinase family members, Plk1 is the most extensively studied. It primarily plays a role in mitosis, thus functionally closest to that of Polo in Drosophila and Cdc5 in the budding yeast. On the other hand, the functions of other Plk members seem to be more diverse and much less understood [17,18,21,24-29]. Extensive studies in the past have shown that Plk1, Plk3, and Plk4 are involved in the genesis and/or development of tumors [30-34].

Murine Plk3 was initially identified as a fibroblast growth factor-inducible kinase (Fnk) in NIH 3T3 cells in a differential display study [35]. Human Plk3 was later cloned and named as Prk (**p**roliferation-**r**elated **k**inase) [33]. The amino acid sequence of human Plk3 shares 36% and 33% overall identity to Plk1 and Polo, respectively [33]. Plk3 has the structure that is typical of a Plk family member, with an N-terminal kinase domain and a C-terminal PBD with two Polo boxes [21] (Figure 1).

The Plk3 mRNA level was found to be inducible by mitogen stimulation with peak expression at the G_1 _phase, whereas its protein level seems to be fairly constant in mitogen-stimulated cells throughout the cell cycle [25,36,37]. However, a more recent report, shows that Plk3 protein level is also cell cycle-regulated with a pattern similar to that of its mRNA [38]. Plk3 is considered an immediate early response gene, whose expression does not depend on *de novo *protein synthesis [25,33,35]. The kinase activity of Plk3 has also been shown to oscillate during the cell cycle and it is subjected to regulation by a variety of stress conditions, including genotoxic insults, hypoxic treatment, and osmotic shock [27-29,36,39-41].

The function of Plk3 appears to be rather diverse. Plk3 is involved in multiple phases of the cell cycle [38,42]. Plk3 is closely associated with spindle poles, mitotic spindles, and midbody during mitosis [43]. Plk3 overexpression induces cell cycle arrest at the M phase followed by apoptosis, which likely results from deregulated microtubule dynamics and centrosomal function [43]. Plk3 may also regulate the onset and/or progression of mitosis and meiosis [44]. Plk3 phosphorylates and regulates the subcellular localization of Cdc25C phosphatase [44,45], a key regulator of the G_2_/M transition [46]. Plk3 is reported to mediate the G_1_/S transition and appears to be required for the S phase entry [38,47]. In addition, Plk3 phosphorylates proteins that are important for regulating DNA replication, such as topoisomerase IIα and DNA polymerase δ (Pol δ) [42,48,49]. Furthermore, Plk3 regulates Golgi fragmentation during mitotic entry in mammalian cells [50-52].

**Figure 2:**
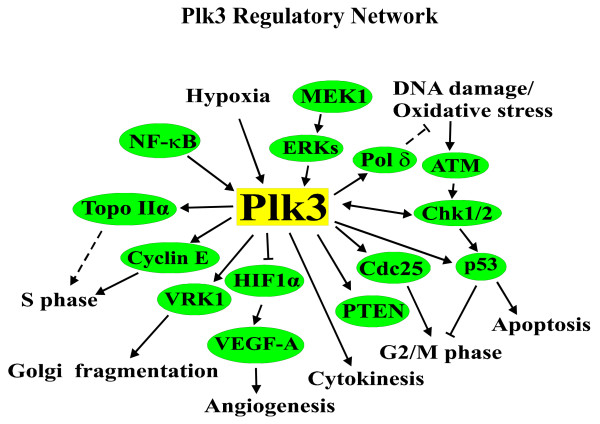
**Known functions of Plk3**. Plk3 regulates a number of cellular activities by modulating a wide array of cellular components. Arrows indicate activation, T bars indicate inhibition.

Plk3 also has a role in apoptosis and stress responses. Ectopic expression of Plk3 causes cell cycle arrest followed by chromatin condensation and apoptosis in cultured cells [39,43,53,54]. Plk3 is activated by cellular responses to DNA damage that produces ROS [27-29,36,39,40,55]. Genotoxic agents such as hydrogen peroxide (H_2_O_2_), ionizing radiation, methylmethane sulfonate, ultraviolet light, and adriamycin strongly activate Plk3 [27-29,36,39,40,55], which in turn phosphorylates p53 at Ser-20 [55]. Ser-20 phosphorylaiton is known to be associated with the stability of p53 [56]. Thus, Plk3 may function to reinforce DNA damage checkpoint responses or to preferentially mediate certain types of DNA damage insults. Plk3 also directly regulates Chk2 and vice versa [36,40]. These results underscore the dynamic role of Plk3 in the DNA damage checkpoint control. Activation of Plk3 by hypoxia and osmotic stress has also been reported in certain cell type [27,28]. Despite the diverse functions of Plk3 discovered in various cell-based in vitro assays, *PLK3 *null mice develop rather normally and are fertile [19]. It is likely that the lack of noticeable phenotype is a result of compensatory mechanisms at the whole animal level. Figure 2 summarizes known functions of Plk3.

**Figure 3:**
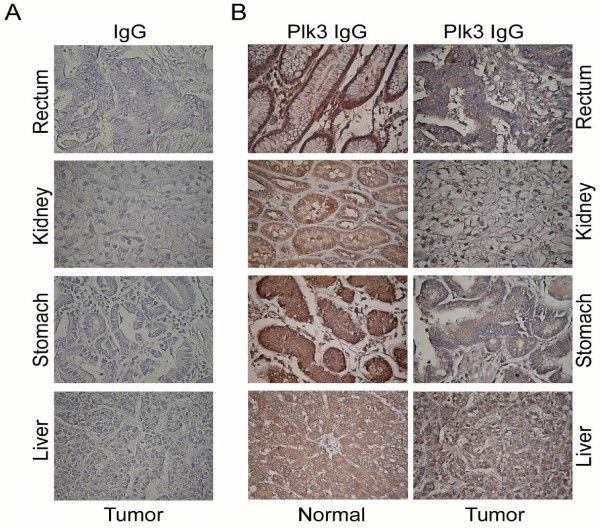
**Plk3 expression in selected human tumors**. Immunohistochemistry was performed on paraffin sections of indicated human tumor and corresponding normal tissue samples using the Plk3 antibody. IgGs were used as control.

**Figure 4:**
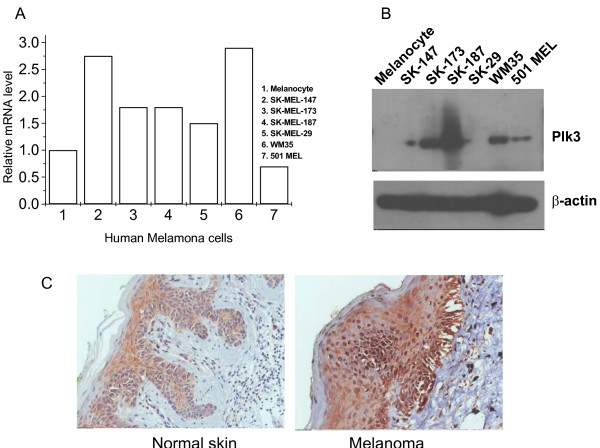
**Plk3 mRNA and protein expression in selected human melanoma cell lines and tissue samples**. A. Real-time PCR was performed on RNA samples from normal melanocytes and selected human melanoma cell lines with DNA primers for Plk3. B. Western blot was performed on normal melanocytes and selected human melanoma cell lines with Plk3 and β-actin antibodies. C. Immunohistochemistry was performed on paraffin sections of human melanoma specimens and corresponding normal skin tissues using the Plk3 antibody.

Plk3 expression is often deregulated in human cancers. Reduced mRNA levels of *PLK3 *in many types of cancer has been reported, including lung cancer, head and neck cancer, and colon cancer [33,34,57], suggesting that reduced Plk3 expression may contribute to tumor development. Consistent with these early studies, we have demonstrated that Plk3 protein levels are also reduced in several other human malignancies including those of kidney, liver, stomach, and rectum (Figure 3). Moreover, we have observed that expression of Plk3 mRNA and protein is significantly deregulated in human melanoma cell lines and tumor tissues (Figure 4). In fact, Plk3 has been generally regarded as having a tumor-suppressing function [30,58]. This notion has been supported by a mouse genetic study showing that, compared with wild-type littermates, *PLK3 *null mice are prone to the development of malignancies in several organs later in the life [19]. The lower levels of *PLK3 *mRNA in cancer cells appear to be a result of reduced mRNA transcription [33]. Of note, mutations of *PLK3 *coding sequences seem to be rare, at lease in lung cancers [59]. Thus, the expression level of Plk3 may play a major role in Plk3's association with cancer development. It is also noteworthy to point out that the *PLK3 *gene is located at chromosome 1p34 where a high frequency of loss of heterozygosity occurs in many human cancers. This implies its close relationship between the loss of its function and tumorigenesis [34,60,61].

## Plk3 and tumor angiogenesis

### Plk3 regulates tumor angiogenesis through HIF-1α

Neovascularization as the result of angiogenesis is essential for progression of solid tumor as the fast growing tumor mass needs to be supported by oxygen and nutrients from the blood [1,2]. One of the key events that triggers tumor angiogenesis is cellular hypoxia, which is a consequence of blood restriction in the inner portion of a solid tumor [2,3,8]. Thus, cellular responses to hypoxia play a pivotal role in tumor angiogenesis [2,3,8].

HIF-1 is the primary player in cellular hypoxic responses and the initiating factor for tumor angiogenesis [1,9,10]. HIF-1 is an oxygen sensing transcription factor containing a constitutively expression β subunit (HIF-1 β) and an inducible α subunit (HIF-1α) [1,10,11,62]. Upon activation by hypoxia, HIF-1 trans-activates an array of genes whose products in turn drive the cellular machinery for hypoxic responses [1,10,11]. Among the most important HIF-1 inducible proteins are VEGF, fibroblast growth factor (FGF), and glucose transporter [1,2]. These proteins are known to stimulate the formation of vasculature or nutrient transport [1,2].

**Figure 5:**
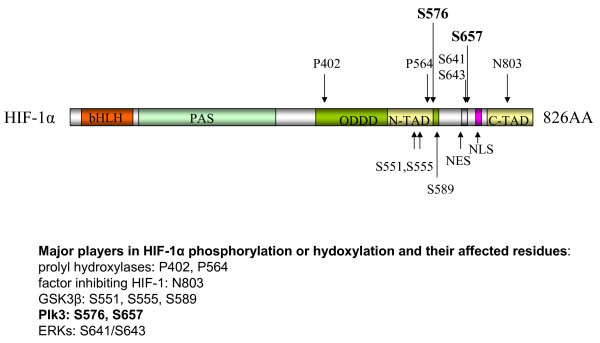
**Domain structure of human HIF-1α, showing Plk3 phosphorylation sites**. Human HIF-1α contains a basic helix-loop-helix domain (bHLH), a PAS domain, an oxygen-dependent degradation domain (ODDD), an N-terminal transactivation domain (N-TAD), and a C-terminal transactivation domain (C-TAD). The hydroxylation sites (P402, P564, and N803) are indicated. The Plk3 phosphorylation sites (S576, S657) are bolded. NES: nuclear exporting signal. NLS: nuclear localization signal.

The activity of HIF-1 is controlled by the availability of its α subunit (HIF-1α), which is primarily regulated at the post-translational level [10,11]. HIF-1α is subjected to multiple types of covalent modifications including hydroxylation, phosphorylation, acetylation, and ubiquitination [10,11]. Although most of these modifications are associated with HIF-1α stability, hydroxylation and ubiquitination are the primary mechanisms by which the HIF-1α protein level is negatively controlled within the cell [10,11]. In normoxia, HIF-1α is hydroxylated by prolyl hydroxylases at two proline residues (Pro-402 and Pro-564 in human) and by factor inhibiting HIF-1 at the asparagine residue (Asn-803) [10,11] (Figure 5). HIF-1α hydroxylation facilitates its association with ubiquitin E3 ligase pVHL and subsequent ubiquitination that leads to HIF-1α degradation by the proteosome [10,11]. Under hypoxic condition, HIF-1α hydroxylation is inhibited, resulting in reduced ubiquitination with concomitant stabilization of HIF-1α protein. In turn, the elevated HIF-1α, in conjunction with HIF-1β, promotes expression of HIF-1-responsive genes [10,11].

The association of Plk3 with tumor angiogenesis was discovered in a recent genetic study demonstrating that *PLK3 *null mice display an increased tumor incidence [19]. Importantly, tumors developed in these mice are frequently large in mass and more vasculated [19]. Further biochemical analysis revealed that Plk3 modulates HIF-1α protein levels in response to hypoxic conditions [17,19]. Specifically, compared with wild-type mouse embryonic fibroblasts (MEFs), *PLK3 *null MEFs exhibit an elevated level of HIF-1α and a hyper-sensitive response to the treatment with hypoxia or with nickel ions that elicit hypoxia-like cellular responses [17,19]. Consistently, expression of VEGF-A, a major HIF-1α response protein, is also higher in *PLK3^-/- ^*MEFs than in wild-type MEFs [19]. Ex vivo analyses confirmed the role of Plk3 in regulating the stability of HIF-1α [17,19]. Furthermore, ectopically expressed Plk3 was able to suppress the nuclear accumulation of HIF-1α in HeLa cells [19]. This inhibition of HIF-1α nuclear translocation appears to be dependent on the kinase activity of Plk3 since overexpression of only the Plk3 kinase domain is sufficient to suppress HIF-1α accumulation in the nucleus under hypoxic conditions [19]. These studies strongly suggest that Plk3 regulates tumor angiogenesis through modulating the expression level of HIF-1α.

### Plk3 directly phosphorylates HIF-1α and regulates its stability

Phosphorylation of HIF-1α by several protein kinases has been reported to regulate HIF-1α stability and/or nuclear translocation [12-17,19]. The MAP kinases, ERKs, phosphorylate HIF-1α at residues Ser-641 and Ser-643 [15] (Figure 5). This phosphorylation facilitates translocation of HIF-1α from the cytoplasm to the nucleus, thereby promoting HIF-1-mediated transcriptional activity [15,16]. These phosphorylation sites are located far outside of the nuclear localization signal (NLS) but within the nuclear exporting signal (NES) of HIF-1α, suggesting that phosphorylation of these sites regulates nuclear export rather than import of HIF-1α [16] (Figure 5). Glycogen synthase kinase 3 β (GSK3β) phosphorylates HIF-1α at three serine residues (Ser-551, Ser-555, and Ser-589) located within its oxygen-dependent degradation domain (ODDD) [12] (Figure 5). Phosphorylation at these residues by GSK3β enhances HIF-1α degradation in a pVHL-independent manner, resulting in suppression of the HIF-1 activity [12,13].

A recent study identifies Plk3 as a new player in the regulation of HIF-1α stability through direct phosphorylation [17]. This study was prompted by genetic evidence showing the involvement of Plk3 in tumor angiogenesis and HIF-1α regulation [19]. The most obvious possibility is that Plk3 phosphorylates HIF-1α and destabilizes HIF-1α in a manner similar to that of GSK3β [12,13]. Kinase assays using purified recombinant HIF-1α and Plk3 reveal that Plk3, but not the kinase-dead counterpart, strongly phosphorylates HIF-1α in vitro [17]. HIF-1α phosphorylation by Plk3 results in a significant mobility shift on a denaturing gel [17]. Pull-down and Co-IP analyses confirm that Plk3 interacts with HIF-1α [17]. Two serine residues (Ser-576 and Ser-657) of HIF-1α have subsequently been identified as direct targets of Plk3 by mass spectrometry [17] (Figure 5). These two residues are evolutionarily conserved among higher animals and have not been shown to be targets of any other kinases [17]. Ser-576 is located within ODDD whereas Ser-657 is right downstream of the NES of HIF-1α (Figure 5), suggesting that Plk3 can potentially regulate both HIF-1α degradation and its nuclear localization. Further analyses reveal that recombinant HIF-1α proteins containing mutations of these residues were much more stable when they are ectopically expressed in cells [17]. Specifically, a single mutation that replaces either Ser-576 or Ser-657 with alanine greatly stabilizes the HIF-1α protein level when the mutant is expressed in HEK293 cells [17]. Replacing both residues with alanines further stabilizes HIF-1α [17], underscoring the importance of phosphorylation of these residues in controlling HIF-1α stability. It is worthwhile to note that mutation of Ser-576, which is located within the ODDD of HIF-1α, appears to be more effective in stabilizing HIF-1α than mutation of Ser-657. These results are consistent with the previous finding that phosphorylation of residues in this domain by GSK3β compromises HIF-1α stability [12]. Similar to that of GSK3β, the Plk3-dependent destabilization of HIF-1α also appears to be hydroxylation- and pVHL-independent as mutations of Plk3-phosphorylation sites can further stabilize HIF-1α with both key proline residues for hydroxylation mutated to alanines [12,17].

### Plk3 indirectly regulates HIF-1a through PTEN phosphatase

#### PTEN and tumor angiogenesis

The lipid phosphatase and tensin homologue (PTEN) is an important tumor suppressor that is mutated or deleted in many cancers [63,64]. PTEN inhibits the phosphatidylinositol 3-kinases kinase (PI3K) signaling pathway by dephosphorylating the phosphoinositides on the 3' position of the inositol ring, thereby reversing the effect of PI3K leading to activation AKT [65,66]. The close tie between PTEN and tumor angiogenesis has been well established [67-71]. PTEN is capable of suppressing tumor angiogenesis in vivo in multiple tumors [67-71]. The inhibitory effect of PTEN on tumor angiogenesis is through the inhibition of the PI3K signaling cascade leading to activation of AKT [67-71]. The PI3K pathway is known to facilitate tumor angiogenesis by increasing the HIF-1α level [5,11,68]. Accordingly, growth factors and cytokines, such as EGF and insulin, increase HIF-1α protein level [5], since these factors activate the PI3K pathway [66]. AKT may increase HIF-1α through its downstream targets such as mTOR or GSK3β, which regulate the protein synthesis and stability of HIF-1α, respectively [5,11,12]. A more recent study provided direct evidence that GSK3β directly phosphorylates HIF-1α and promotes its degradation in a hydroxylation-independent manner [12] (see previous section). In the PI3K signaling cascade, AKT directly phosphorylates GSK3β at Ser-9, thereby inhibiting its activity [72-74]. Thus, PTEN inhibits tumor angiogenesis by suppressing HIF-1 activity through the reduction of the HIF-1α protein level. This is accomplished by the inhibition of the PI3K pathway that leads to lower GSK3β activity.

#### Regulation of PTEN by phosphorylation

**Figure 6:**
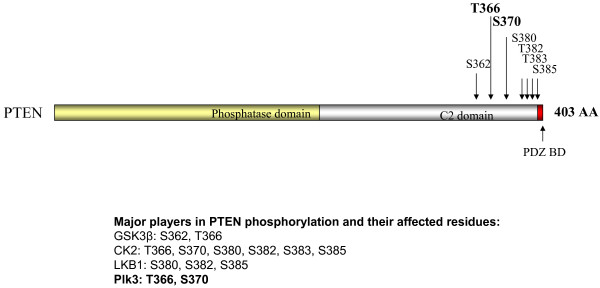
**Domain structure of human PTEN, showing Plk3 phosphorylation sites**. Human PTEN contains an N-terminal phosphatase domain, a C-terminal C2 domain, and a PDZ binding domain. Major phosphorylation sites at the C-terminal region are indicated. Plk3 phosphorylation sites are bolded.

PTEN is subjected to phosphorylation by multiple kinases [18,75-82] (Figure 6). Phosphorylation of PTEN affects PTEN activity and stability [18,75-77,79-85]. Most of the identified PTEN phosphorylation sites are located at the C-terminal region of the protein [18,75-77,79] (Figure 6). The C-terminal domain is thus considered the regulatory domain of PTEN [75,86,87]. Casein kinase 2 (CK2) seems to be the major kinase that phosphorylates PTEN [75-77]. The two major sites phosphorylated by CK2 are Ser-370 and Ser-385, although to a lesser extent, Thr-366, Ser-380, Ser-382, and Ser-383 [75-77]. Ser-385, in conjunction with one of the preceding serine sites (Ser-380 or Ser-382, and Ser-385) has also been proposed to be a target of liver kinase B1 [79]. GSK3β has been shown to phosphorylate PTEN at Ser-362 and Thr-366 [78] (Figure 6). Interestingly, the phosphorylation of Thr-366 is strongly enhanced by prior phosphorylation of CK2, suggesting that there is cooperative mechanism between different kinases in PTEN phosphorylation [78]. PTEN phosphorylation by Src tyrosine kinase, a microtubule-associated kinase, and RhoA associated kinase has also been reported [80-82]. A most recent study identified Plk3 as a PTEN kinase that phosphorylates Thr-366 and Ser-370 [18] (Figure 6).

The effect of PTEN phosphorylation on PTEN function appears to be multifaceted. Phosphorylation of Ser-370 and Ser-385 by CK2 has been reported to suppress the PTEN activity [76,77]. So does the tyrosine phosphorylation by Src [81]. In contrast, mutations of Ser-380, Ser-382, and Ser-383 destabilize PTEN, suggesting that phosphorylation of these sites may increase its stability [85,88]. Phosphorylation of PTEN by CK2 at Ser-370 and Ser-385 and by Plk3 at Thr 366 and Ser-370 also stabilize PTEN [18,77]. Interestingly, deletion of the tail-domain or mutation of Ser-380, Ser-382, and Ser-383 greatly reduces the stability PTEN but activates its phosphatase activity [84,88]. A model has been proposed that upon phosphorylation, the C-terminal domain, stabilizes the protein due to a change in protein conformation that inhibits its phosphatase activity [84,87,88]. Dephoshorylation of PTEN releases the inhibitory conformation and renders PTEN more active [84,87,88]. However, a change in the overall PTEN level can have great biological consequences [89]. For instance, haploinsufficiency of PTEN also results in enhanced tumor development [90,91], underscoring the importance of the PTEN protein level in controlling cell proliferation and malignant transformation. Given the fact that PTEN is phosphorylated by multiple protein kinases, it is conceivable that a correct combination of phosphorylation is needed to fine-tune the activity or level of PTEN for a given physiological state.

#### Plk3 phosphorylates and regulates PTEN stability

*PLK3 *ablation also results in significantly increased activity of the PI3K signaling pathway. Specifically, enhanced phosphorylation of Ser-473 of AKT, Ser-9 of GSK3β, and Ser-2448 of mTOR were observed in *PLK3 *null mouse embryonic fibroblasts (MEFs) compared to wild-type MEFs [18]. Further analyses on the upstream components of the PI3K pathway revealed that *PLK3 *null MEFS contained lower levels of PTEN protein [18]. These findings raised the possibility that Plk3 may directly regulate PTEN by phosphorylation. In vitro kinase assays using recombinant PTEN and Plk3 confirmed that Plk3 indeed strongly phosphorylates PTEN [18]. Mass spectrometric analysis identified Thr-366 and Ser-370 as residues that were phosphorylated by Plk3 [18]. The phosphorylation of these two sites by Plk3 was further confirmed using a phospho-specific antibody that recognized phospo-Thr-366 and phospo-Ser-370 [18].

Phosphorylation of these two sites was found to affect the stability of PTEN. PTEN constructs with Thr-366 and Ser-370 mutated to alanines were less stable when transfected into HEK 293 cells [18]. This result is therefore consistent with the reduced endogenous PTEN protein level in *PLK3 *null MEFs [18]. The lower PTEN level in *PLK3 *null MEFs corresponds to a reduced overall PTEN activity as phosphorylation of phosphoinositide-dependent protein kinase 1 (PDK-1) at Ser-241, an indirect target of PTEN [92], was enhanced in *PLK3 *null MEFs [18]. Thus, phosphorylation of Thr-366 and Ser-370 by Plk3 functions to stabilize PTEN, resulting in an increased overall PTEN phosphatase activity in the cell. Of note, these two sites have been reported to be the targets of CK2 [76,77]. Thr-366 can also be phosphorylated by GSK3β [78]. Therefore, Thr-366 and Ser-370 of PTEN appear to be subjected to modification by multiple protein kinases.

PTEN stability affected by Plk3 phosphorylation appears to be proteosome-dependent since proteosome inhibitor was able to increase PTEN protein level in *PLK3 *null MEFs [18]. This is consistent with previous findings that PTEN is subjected to ubiquitination and degradation by the proteosome [85,93,94]. Notably, there is considerable phosphorylation of Ser-370 and Thr-366 of PTEN at the resting state that is readily detectable by the phospho-specific antibody [18]. This constitutive phosphorylation is partially inhibited by LiCl, an inhibitor of GSK3β and CK2 [95], supporting the previous findings that one or both of these two kinases phosphorylate these two sites [76,77].

**Figure 7:**
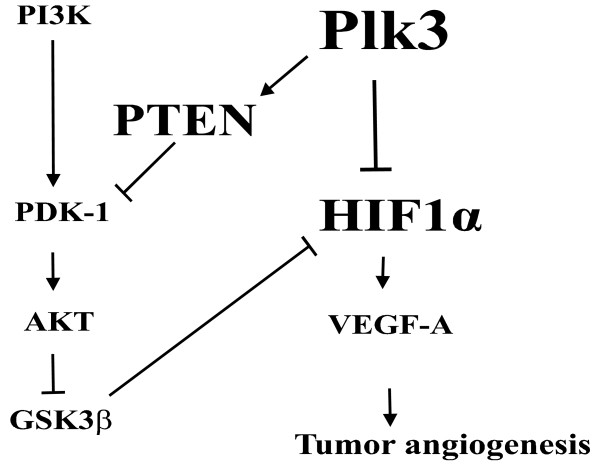
**Proposed model for Plk3 regulation of HIF-1α**. Plk3 directly phosphorylates HIF-1α and facilitates its degradation. Plk3 also phosphorylates PTEN and stabilizes it, thereby increasing overall PTEN activity. PTEN inhibits the PI3K pathway and leads to activation of GSK3β, which phosphorylates and destabilize HIF-1α. Inhibition of HIF-1α by Plk3 through direct and indirect mechanisms results in reduced tumor angiogenesis.

Given the known effect of the PI3K pathway on HIF-1α stability and the effect of Plk3 on PTEN, it is conceivable that Plk3 may affect HIF-1α stability and HIF-1 activity indirectly through regulation of the PI3K signaling pathway. Combined, these studies yield an attractive model for Plk3 regulation of HIF-1α stability/HIF-1 activity both directly by phosphorylating HIF-1α and indirectly by phosphorylating PTEN (Figure 7). This model nicely explains that the function of Plk3 has in the suppression of tumor angiogenesis, as well as tumor development in vivo.

## Plk3 as a potential therapeutic target for cancer treatment

The newly discovered role of Plk3 in tumor angiogenesis highlights its potential as a therapeutic target for cancer treatment. Tumor angiogenesis has been well established as a valuable cellular process for drug intervention [2,6,7]. Protein kinases have been favored by the pharmaceutical industry for cancer drug development [96]. Screening small chemicals that interfere with the ATP binding capacity of kinases thereby inhibiting their kinase activities is the conventional approach for identifying drug leads [96,97]. Among the Plk family kinases, Plk1 has been the major focus of drug development [58,98,99]. Several ATP-competitive and non-ATP competitive compounds have been identified as having the potential to inhibit Plk1 activity [58,98,99]. These compounds possess the ability, albeit highly variable, to slow cell proliferation of many cancer cell lines [58,98,99]. Some of these compounds have been tested for their efficacy in clinical trials for the treatment of cancers of various origins [58,98,99]. Many Plk1 inhibitors also block Plk3 activity to a certain extent [58]. However, in contrast to Plk1, which plays a positive role in cell proliferation and tumor development, Plk3 is regarded as having a tumor-suppressing function [21,30]. The latest study on the effect of Plk3 on tumor angiogenesis has provided results that are consistence with this notion [18]. It is conceivable that a chemical compound that inhibits Plk3 activity specifically might have no beneficial effect to cancer treatment or management. Therefore, it is likely that the anti-tumor effect of those compounds which have an activity toward both Plk1 and Plk3 may result from Plk1 inhibition. Consequently, the discovery of Plk3 activators would be a more rational approach for anti-cancer drug development.

Targeting Plk3-mediated tumor angiogenesis may offer several advantages over Plk1 in cancer treatment. It have been shown that Plk1 knock-down via RNAi results in cell cycle arrest followed by apoptosis and that Plk1 knock-out mice is embryonically lethal [32,100]. These results suggest that Plk1 is vital for normal cell proliferation and survival. Consequently, inhibition of Plk1 is likely to have a detrimental effect on normal cells in addition to cancer cells. In contrast, *PLK3 *ablation in mice does not noticeably perturb animal development except for enhanced tumor angiogenesis in mice of advanced age [19]. Therefore targeting Plk3 would likely to have less side-effect *in vivo*. In addition, therapeutic intervention that activates Plk3 should lead to enhanced apoptosis in targeting cells as the effect has been observed in cells that overexpress Plk3 [21].

## Concluding remarks

In spite of extensive research in the past which has yielded a sizable body of knowledge about the biochemical and cellular functions of Plk3, it remains as a protein kinase we know very little about, especially with regard to its physiological and patho-physiological roles. Mouse genetic studies have yielded valuable information about Plk3's role in suppressing tumor angiogenesis through negative regulation of the PI3K signaling network and the HIF-1 activity. Additional analyses are clearly needed to fully understand the physiological role of *PLK3 *in animal development and stress responses. Given its documented roles in suppressing cell proliferation *in vitro *and tumor development *in vivo*, Plk3 represents an attractive target for the development of anti-cancer compounds.

## Competing interests

The authors declare that they have no competing interests.

## Authors' contributions

DX: Designed and executed experiments and also involved in writing the manuscript. QW: Designed and performed experiments. YJ: Conceived experiments and interpreted data. YZ: Carried out experiments. EV-SM: Carried out experiments. IO: Data interpretation and discussion. WD: Direct the study; data interpretation, manuscript writing. All authors read and approved the final manuscript.
